# Pyruvate dehydrogenase enzyme dipstick test in traumatic brain injury: A concern

**DOI:** 10.4103/0974-2700.58650

**Published:** 2010

**Authors:** Viroj Wiwanitkit

**Affiliations:** Wiwanitkit House, Bangkhae, Bangkok 10160, Thailand

Sir,

I read the article by Sharma *et al*. with great interest.[1] Sharma *et al*. report on a new alternative method of measurement of pyruvate dehydrogenase enzyme by dipstick test in traumatic brain injury and mentions that this alternative has many advantages over the classical spectrophotometric method.[1] Pathophysiologically, traumatic brain injury can induce expression and phosphorylation of pyruvate dehydrogenase, the specific rate-limiting enzyme coupling cytosolic glycolysis to the mitochondrial citric acid cycle.[2] Since the main function of pyruvate dehydrogenase is the maintenance of homeostasis of brain glucose metabolism, dysregulated glucose metabolism may be seen in traumatic brain injury.[2] Measurement of pyruvate dehydrogenase can be a useful laboratory investigation in the management of patients with traumatic brain injury. In laboratory medicine, the standard method for measurement of pyruvate dehydrogenase is based on the principle of enzymatic measurement[3] and the spectrophotometric method is generally used.[3] However, to perform a spectrophotometric measurement of the enzyme, a standard medical laboratory has to be available. Since traumatic brain injury is considered an emergency condition, an alternative method that can be used as a bedside or point-of-care test is required. In the paper by Sharma *et al*.,[1] a new dipstick test was used and showed good results. However, there is a need to consider for the ability in laboratory diagnosis which include sensitivity, specificity, accuracy, predictive value and etc. Indeed, a complete evaluation of this new test has to be done. The diagnostic sensitivity and range of analysis (upper and lower limit for diagnosis) have to be tested. It is possible that the dipstick test might produce results that deviate from that obtained with the gold standard. This should be verified. In addition, based on the nature of dipstick, the pre-analytical error due to variation in the dipping technique is an important practical point that needs consideration. Due to the described reasone, a careful practice is needed for applying the test, dipping the test strip, into the specimen. The clinical diagnostic capability (sensitivity, specificity, and positive and negative predictive values) of this test in actual clinical practice is still to be established. In addition, the question of the cost-effectiveness of this alternative method as compared to the classical method has to be considered. This data is needed although Sharma *et al*. have given the direct cost of each test in their paper. These data will be useful for further decision making regarding the use of the new dipstick test.

## References

[1] Sharma P, Benford B, Li ZZ, Ling GS (2009). Role of pyruvate dehydrogenase complex in traumatic brain injury and measurement of pyruvate dehydrogenase enzyme by dipstick test. J Emerg Trauma Shock.

[2] Xing G, Ren M, Watson WA, O'Neil JT, Verma A (2009). Traumatic brain injury-induced expression and phosphorylation of pyruvate dehydrogenase: a mechanism of dysregulated glucose metabolism. Neurosci Lett.

[3] Wilkinson JH (1972). Quantitative measurement of enzymes. Ann Clin Lab Sci.

